# Modeling the effectiveness of RT-PCR, RT-LAMP, and antigen testing strategies for COVID-19 control

**DOI:** 10.1186/s12879-025-11793-7

**Published:** 2025-10-17

**Authors:** Tanakorn Chantanasaro, Chayanin Sararat, Noppamas Yolai, Pikkanet Suttirat, Kawin Nawattanapaiboon, Somchai Chauvatcharin, Sudarat Chadsuthi, Charin Modchang

**Affiliations:** 1 Biophysics Group, Department of Physics, Faculty of Science, Mahidol University, Bangkok, 10400 Thailand; 2 Center for Disease Modeling, Faculty of Science, Mahidol University, Bangkok, 10400 Thailand; 3 Zenostic Co., Ltd, Bangkok, 10400 Thailand; 4 School of Materials Science and Innovation, Faculty of Science, Mahidol University, Bangkok, 10400 Thailand; 5 Department of Biotechnology, Faculty of Science, Mahidol University, Bangkok, 10400 Thailand; 6 Department of Physics, Faculty of Science, Naresuan University, Phitsanulok, 65000 Thailand; 7 Centre of Excellence in Mathematics, MHESI, Bangkok, 10400 Thailand; 8 Thailand Center of Excellence in Physics, Ministry of Higher Education, Science, Research and Innovation, 328 Si Ayutthaya Road, Bangkok, 10400 Thailand

**Keywords:** COVID-19 testing strategies, Outbreak control, Daily screening, Symptom-based testing, Contact tracing, RT-PCR, RT-LAMP, Antigen tests

## Abstract

**Background:**

The COVID-19 pandemic has highlighted the crucial role of testing in mitigating disease transmission. This study evaluates the effectiveness and cost-efficiency of various testing strategies, including daily screening, symptom-based testing, and contact-based testing, using RT-PCR, RT-LAMP, and antigen tests.

**Methods:**

We employed stochastic modeling on a contact network to assess the impact of these strategies on outbreak control. Simulations were conducted to evaluate the probability of an outbreak, epidemic size, and testing costs for each strategy. Scenarios with varying levels of population immunity were also explored.

**Results:**

Daily screening, particularly with RT-PCR and RT-LAMP, significantly reduced transmission risks but incurred higher costs. Symptom-based testing offered a more cost-effective alternative, albeit with lower efficacy in mitigating outbreaks. Antigen tests, despite their lower sensitivity, proved to be a cost-effective option for symptom-based testing. Turnaround time of symptom-based testing was a more critical factor than assay sensitivity in containing outbreaks. Combining symptom-based testing with contact tracing further reduced outbreak probability. In scenarios with pre-existing population immunity, testing all symptomatic individuals was the most effective and cost-efficient approach when compared to testing a lower proportion of symptomatic individuals.

**Conclusions:**

Our findings suggest testing strategies could be adapted based on the stage of the epidemic, population immunity, and available resources. Daily screening is most effective but costly, while symptom-based testing combined with contact tracing offers a more cost-effective approach. Antigen tests can be a viable alternative for symptom-based testing in resource-limited settings. Rapid case identification and isolation are crucial for optimal outbreak control. These findings provide valuable insights for designing targeted interventions to protect communities while managing limited resources during current and future infectious disease outbreaks.

**Supplementary Information:**

The online version contains supplementary material available at 10.1186/s12879-025-11793-7.

## Background

The Coronavirus disease 2019 (COVID-19) pandemic has had a devastating global impact, with over 775 million cases and approximately 7 million deaths reported worldwide as of May 2024 [1]. The pandemic has triggered a multifaceted crisis, adversely affecting the economy, tourism, and public health and exacerbating poverty [2–8]. In response to this unprecedented challenge, many countries implemented non-pharmaceutical interventions (NPIs) such as physical distancing, remote work, face mask-wearing, and school and workplace closures while awaiting the widespread distribution of effective vaccines [9–13]. However, despite the availability of vaccines, the emergence of new variants and waning immunity has led to ongoing breakthrough infections and reinfections, contributing to a persistently challenging situation [14–18]. Consequently, NPIs remain important in helping to limit transmission and to reduce the impact of the pandemic.

Among the various NPIs, testing and isolating infected individuals has played an important role in mitigating the burden of the COVID-19 outbreak. The rapid identification of infections through testing helps isolate infectious individuals quickly, thereby limiting further transmission from primary cases and potentially reducing subsequent cases [12, 19, 20]. Moreover, screening tests aimed at detecting asymptomatic or pre-symptomatic infectious individuals within the population can significantly help curtail onward transmission [20–22]. By identifying and isolating infected individuals who may not exhibit symptoms, screening tests can break the chain of transmission and prevent the silent spread of the virus in the community.

Several approaches are employed in viral testing to detect the presence of SARS-CoV-2 [23–25]. These approaches involve identifying specific viral components, such as the ribonucleic acid (RNA) or antigens, within an individual’s body to determine current or recent infection with SARS-CoV-2. Nucleic acid amplification testing (NAAT), which includes the widely used reverse transcription polymerase chain reaction (RT-PCR), is one such approach that detects viral RNA genes [23, 24]. In fact, RT-PCR is considered the gold standard for COVID-19 diagnosis due to its high sensitivity and specificity, making it an essential tool for accurate case detection and outbreak management. However, RT-PCR tests often require specialized equipment and trained personnel, which can limit their availability and increase costs. In contrast, rapid antigen tests or antigen test kits (ATK) target specific viral proteins known as antigens and are often used for rapid, point-of-care testing [23, 24]. ATK can detect the presence of viruses in a clinical sample within 30 min without the need for a complex laboratory setup or specialized personnel [26]. Although antigen tests may have lower sensitivity compared to RT-PCR, they offer several advantages, such as cost-effectiveness, faster turnaround times, and ease of use, making them suitable for large-scale screening and rapid outbreak control [23, 24, 27]. Another promising testing method is reverse-transcription loop-mediated isothermal amplification (RT-LAMP), which is a reliable and rapid screening test that can be used in the field or under non-laboratory conditions [28]. RT-LAMP has been shown to have high sensitivity and specificity, comparable to RT-PCR while being more cost-effective and easier to implement in resource-limited settings [25, 28].

Modeling studies have suggested that effective testing strategies, when combined with other interventions such as contact tracing, isolation, and quarantine, have the potential to prevent both the initial epidemic and its resurgence [20, 29–32]. Several studies have examined the impact of different testing approaches on transmission dynamics. For instance, Litwin et al. developed an individual-based model to assess various testing strategies in mental health hospitals. Their findings indicate that active testing methods, such as entry screening and routine testing (once-weekly or twice-weekly), are more effective in reducing outbreak probability than symptom-based testing alone [33]. Similarly, Cui, Ni, and Shen simulated transmission dynamics on a complex network with different testing strategies. They found that increasing daily testing volume and implementing testing more rapidly can help lower the infection peak [34].

Our work builds upon and extends previous literature in several important ways. While Larremore et al. [35] demonstrated that testing frequency and turnaround time are more critical than test sensitivity for population screening, our study specifically addresses how different testing modalities (RT-PCR, RT-LAMP, and antigen testing) perform when implemented in diverse testing strategies (daily screening, symptom-based, and contact-based). Unlike Hart et al. [36], who focused on antigen testing in a multi-scale modeling framework to estimate outbreak risk, our study provides a comprehensive comparison of multiple testing technologies across different implementation strategies while accounting for cost-effectiveness. Additionally, whereas Gostic et al. focused primarily on travel-related symptom and risk screening, our analysis examines testing strategies in broader community contexts and provides actionable insights for resource-limited settings [37].

This study aims to support public health decision-making across different phases of an epidemic through several specific objectives. In the early phase of an outbreak, our model evaluates strategies to minimize the probability of a major epidemic, which is critical when imported cases are first detected in a previously unaffected community. For established outbreaks, we assess approaches to minimize the epidemic size (total number of infections) while optimizing resource allocation. In resource-limited settings, we evaluate the cost-effectiveness of different testing strategies to identify approaches that provide maximum outbreak control per unit of investment. Additionally, for later epidemic phases with partial population immunity, we examine testing strategies that can prevent resurgence while maintaining operational sustainability. By addressing these distinct decision-support objectives, our modeling provides tailored guidance for different epidemic contexts, resource constraints, and public health goals.

In this study, we employ an individual-based modeling approach to investigate the impact of various testing strategies on mitigating COVID-19 transmission. We compare the effectiveness of daily screening, symptom-based testing, and contact-based testing in reducing outbreak risk and epidemic size. Furthermore, we examine the influence of factors such as turnaround time and assay sensitivity on the efficacy of these testing strategies. To provide a comprehensive understanding of the trade-offs involved, we also conduct a comparative cost analysis for each testing strategy in terms of outbreak control. By evaluating the interplay between testing strategies, their associated costs, and their effectiveness in curbing disease spread, this study aims to offer valuable insights for policymakers and public health authorities in designing optimal testing approaches to combat the ongoing COVID-19 transmission and future infectious disease outbreaks.

## Methods

### Viral dynamics within host body

To construct viral load dynamics, we processed empirical data from Chia et al. [38]. Their study reported cycle threshold (Ct) values from RT-PCR testing, which we converted to viral load (VL) using the established relationship [39]:

1 $$\:VL\left(t\right)={10}^{15.043\:-\:(Ct\:\times\:\:0.296)}.$$

This conversion transforms the available empirical Ct data into viral load values that serve as model inputs. After this preprocessing step, only viral loads *VL*(*t*) are used in all subsequent model calculations; Ct values are not used directly in the simulation.

**Fig. 1:**
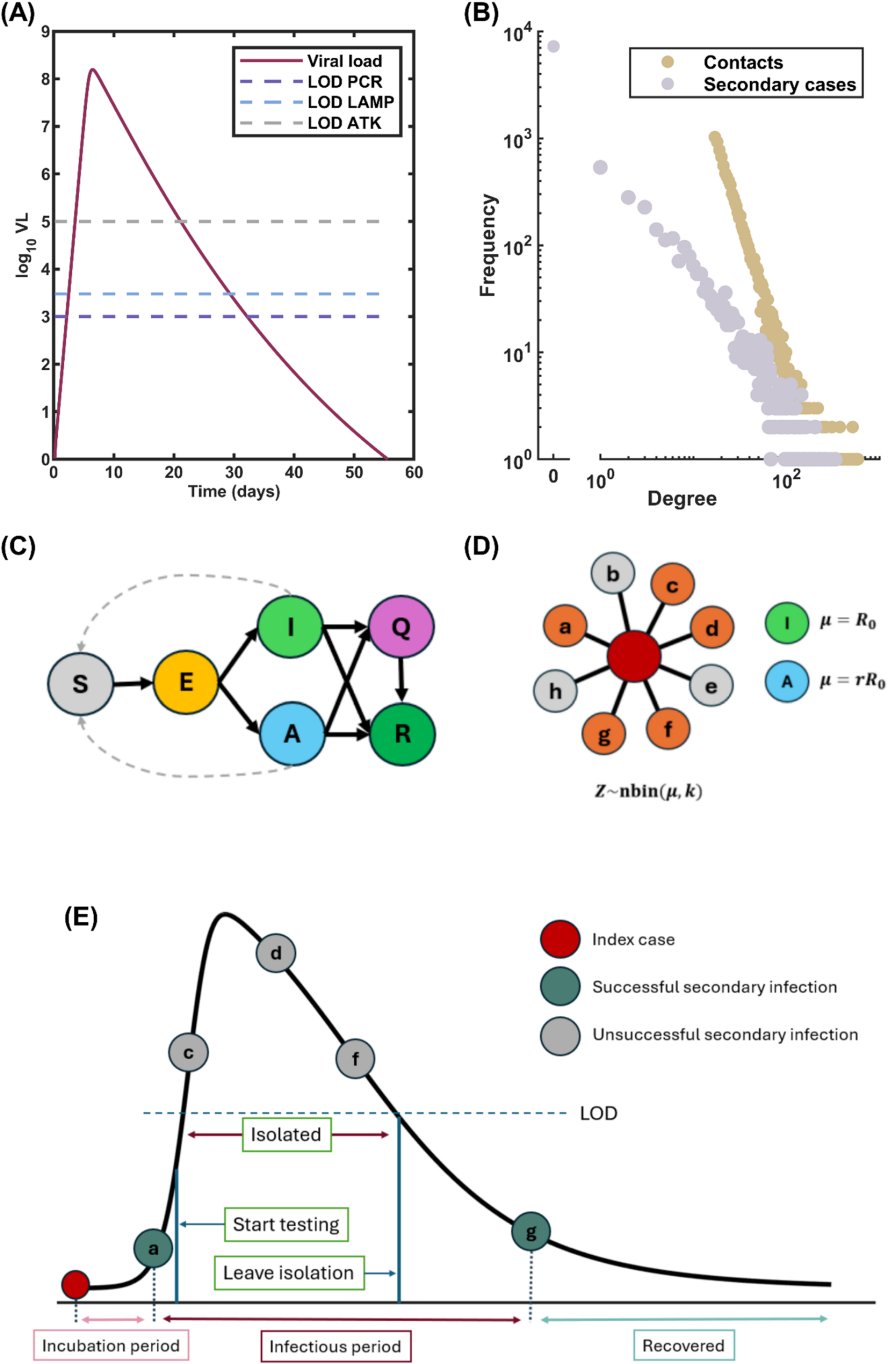
Model Structure. **A** Estimated viral load dynamics of an infectious individual following infection, plotted alongside the limits of detection (LOD) of three testing assays: RT-PCR, RT-LAMP, and ATK. **B** Distribution of the number of contacts in the simulated contact network (yellow dots) and the expected number of secondary cases resulting from an infected individual (gray dots). The contact network is generated using a power-law distribution, capturing the heterogeneity in social interactions. **C** Compartmental model representing the progression of an infected individual’s infection status. Solid arrows depict the transitions between different stages of infection. Dashed arrows indicate the potential for disease transmission from infectious individuals to susceptible ones. **D** The number of secondary infections is drawn from the negative binomial distribution with mean $\:{R}_{0}$ for symptomatic case and $\:{rR}_{0}$ for asymptomatic case, both with the dispersion parameter $\:k$. Individuals highlighted in orange denote contacts that were randomly assigned as secondary infections resulting from exposure to the index case. **E** The isolation of infected cases will be triggered if an infected person has viral load higher than the LOD. The isolated individual will leave the isolation when the viral load is less than the LOD and can still transmit the disease

However, since the Ct values prior to diagnosis were not available in the dataset, we estimated the viral dynamics before diagnosis for an infected individuals by fitting the number of viral copies post-diagnosis to the innate immune response model outlined in [40]. (See supplementary Information and Fig. S1.) The estimated viral load of infected individuals since time of infection $\:t$ is plotted in Fig. 1A showing the best fitted viral load curve. The fitting process was conducted using a nonlinear least-squares function in MATLAB. We obtained the fitted parameters for the innate immune response model, where $\:\beta\:=3.3710\times\:{10}^{-8}$, $\:\delta\:=0.5432$, $\:\pi\:=34.8702$, $\:\varnothing\:=10.1086\times\:{10}^{-8}$, and $\:\rho\:=0.9999$ with a NMSE of 0.029. The viral load over the course of infection (Fig. 1A) was then generated using the estimated outputs. Additionally, we assumed that the viral dynamics of all infected individuals follow this estimation, representing a homogeneous viral progression across the population. This simplifying assumption was made to maintain model tractability while capturing the essential features of transmission dynamics.

## Relative infectiousness profile

We assessed the level of infectivity exhibited by an individual throughout their infection by creating a relative infectiousness profile $\:p\left(t\right)$. This profile was constructed using viral load (VL) values, following the methodology detailed in [40]

2 $$\:p\left(t\right)=\frac{{VL\left(t\right)}^{h}}{{VL\left(t\right)}^{h}+{K}_{m}^{h}},$$

where $\:VL\left(t\right)$ is the estimated viral load as determined in the previous subsection. The constant $\:h$ and $\:{K}_{m}$ are fixed at specific values, with $\:h$ set to 0.51 and $\:{K}_{m}$ set to 8.9$\:\:\times\:\:$10^6^ RNA copies/mL, respectively.

The relative infectiousness profile *p*(*t*) quantifies the time-varying transmission potential of an infected individual throughout their infection course. In our model, transmission events occur stochastically based on this profile. Specifically, when an individual becomes infected, we first determine the total number of secondary infections (*Z*) they will generate based on a negative binomial distribution with mean *R*₀ (for symptomatic cases) or *rR*₀ (for asymptomatic cases) and dispersion parameter *k*. For each infected individual who generates *Z* secondary infections, we then randomly sample *Z* transmission times from a probability distribution proportional to the infectiousness profile *p*(*t*). This sampling continues until day 33 post-infection, at which point the viral load and corresponding infectiousness become negligible (**Fig. S2**). This approach captures the biological reality that transmission probability varies substantially throughout infection, peaking during days 4–6 when viral load is highest, while allowing for possible transmission events throughout the entire infectious period.

If an infected individual is isolated during any of these predetermined transmission times, those specific transmission events are prevented, thereby reducing the actual number of secondary cases below Z. This stochastic implementation enables our model to realistically simulate the impact of testing and isolation strategies on disease transmission (Fig. 1).

### Contact network

This study simulated the transmission dynamics within a contact network characterized by nodes exhibiting a power-law distribution in their degree. Power-law distributions are pervasive across various intricate systems, such as wealth distribution, city sizes, social networks, word frequencies, and citations [41–45]. Studies have indicated that social interaction networks often adhere to a power-law distribution [46, 47]. Utilizing the Barabási–Albert (BA) algorithm, we constructed the network and subsequently conducted simulations to model the transmission process within it [48]. Nodes and links within the network represent individuals and their respective contacts. The contact network comprises 10,000 nodes, with an average degree of 34 (Fig. 1B). This average degree choice ensures that the number of secondary cases derived from a negative binomial distribution for all individuals remains within the bounds of their actual contacts. The degree distribution of the generated network follows a power-law distribution with the power-law exponent of 2.881.

### Model structure

In the model, each individual can possess one of six states: susceptible $\:\left(S\right)$, latently infected $\:\left(E\right)$, symptomatic infectious $\:\left({I}_{S}\right)$, asymptomatic infectious $\:\left({I}_{A}\right)$, isolated $\:\left(Q\right)$, and recovered $\:\left(R\right)$, as depicted in Fig. 1C. When susceptible individuals become infected, they transition to the latent state and progress through the course of infection. Once in the latent state, there is a probability, denoted as $\:{k}_{asym}$, that they become asymptomatic. The asymptomatic infectious individual has a lower viral load and thus is less infectious than the symptomatic one [49]. A portion of individuals undergo testing to determine their infection status. If a person tests positive for the disease, they are immediately isolated from the general population.

Individuals who test positive are placed in isolation and monitored regularly. They remain isolated until their viral load decreases below the test’s limit of detection (LOD), at which point they are released. Due to the different LODs of each assay, isolation duration varies: individuals initially tested with RT-PCR typically remain isolated until approximately day 34 post-infection, those tested with RT-LAMP until day 31, and those tested with ATK until day 23.

Importantly, individuals released from isolation may still carry a viral load sufficient for transmission, albeit at a lower probability than during peak infectiousness. Our model accounts for this by allowing post-isolation transmission events to occur according to the infectiousness profile. Once an individual has completed all their potential transmission events (either successfully or prevented through isolation), they transition to the recovered state (Fig. 1E). All recovered individuals are assumed to acquire lifelong immunity with no possibility of reinfection.

**Table 1:** Model variables, parameters and their default values

Symbol	Description	Default/Initialization	Source
State variables			
$\:S$	Number of susceptible individuals	$\:S\left(0\right)=N-1$	Assumed
$\:L$	Number of latently exposed individuals	$\:L\left(0\right)=1$	Assumed
$\:{I}_{s}$	Number of symptomatic infectious individuals	$\:{I}_{S}\left(0\right)=1$	Assumed
$\:{I}_{A}$	Number of asymptomatic infectious individuals	$\:{I}_{A}\left(0\right)=1$	Assumed
$\:Q$	Number of isolated individuals	$\:Q\left(0\right)=1$	Assumed
$\:R$	Number of recovered individuals	$\:R\left(0\right)=1$	Assumed
Model parameters			
$\:N$	Number of nodes (population size)	10,000	Assumed
$\langle{k}\rangle$	Mean degree	34	Assumed
$\:{R}_{0}$	Basic reproduction number	5.08	[50]
$\:k$	Dispersion parameter	0.08	[51]
$\:{k}_{asym}$	Probability of being asymptomatic	0.427	[52]
$\:r$	Reduction in infectiousness of asymptomatic individuals	0.58	[49]
	Incubation period	5.1 days	[53]
$\:Z$	Number of secondary infections	$\:\sim {nbin}(\mu\:,k)$	
$\:h$	Hill coefficient	0.51	[54]
$\:{K}_{m}$	half-maximal viral load	8.9$\:\:\times\:\:$10^6^ RNA copies/mL	[54]
Testing proportion	Proportion of individuals tested	0%, 25%, 50%, 75%, and 100%	Assumed

Note: The values presented correspond to default parameter settings and initial conditions, unless otherwise stated

In the transmission network, the number of secondary infections ($\:Z$) resulting from a single primary case in the absence of interventions or susceptible depletion was modeled using the negative binomial distribution with a mean equal to the basic reproduction number $\:\left({R}_{0}\right)$ and a dispersion parameter $\:\left(k\right)$. For the asymptomatic infectious individual, the number of secondary infections was adjusted by a constant $\:r$, which reflects the reduced infectiousness [49]. Therefore, the mean number of secondary infectious individuals from asymptomatic cases is $\:r{R}_{0}$ (Fig. 1D). After drawing $\:Z$ from the negative binomial distribution for each individual in advance of simulating the realized transmission dynamics, we ranked two sets of number (number of contacts and secondary infections) from maximum to minimum, then paired the number with the same ranking order. This assumes that people with a greater number of contacts tend to make more secondary infections than people with less contacts. The time that infected individuals make their secondary infection is drawn from a random number distribution that was distributed according to the infectiousness profile. Therefore, if an infected individual is isolated during the time the secondary infection is supposed to happen, the transmission will not occur. The model parameters and their default values are summarized in Table 1.

Note that our model differs fundamentally from traditional compartmental approaches. Rather than modeling explicit state transitions, individual states emerge from underlying viral load dynamics and transmission events. When infected, each individual receives a viral load trajectory *VL*(*t*), *Z* predetermined secondary infections drawn from NegBin(*µ*, *k*), where *µ* = *R*_0_ (symptomatic) or *rR*_0_ (asymptomatic), and transmission times sampled from the infectiousness profile *p*(*t*).

The traditional latent and infectious states are not explicitly modeled. Instead, an individual is considered latently infected from infection until their first transmission occurs, infectious after their first transmission if it occurs before isolation, isolated when detected with VL exceeding LOD at testing time, and recovered after all potential transmissions complete.

This means the latent period is not a fixed parameter but emerges from viral dynamics. Two simultaneously infected individuals may have different latent periods based on their sampled transmission times. Importantly, early detection and isolation may prevent an infected individual from ever becoming ‘infectious’ in the epidemiological sense, despite harboring virus.

### Integration of model components

Our model integrates viral dynamics, network structure, disease progression, transmission mechanics, and intervention strategies through a unified simulation framework. The viral load trajectory *VL*(*t*) (Eq. (1) and Fig. 1A) serves as the fundamental driver, determining both the infectiousness profile *p*(*t*) (Eq. 2) through a Hill function transformation and the detectability by different assays based on their limits of detection (LOD).

The contact network (Fig. 1B) constrains disease spread, as transmissions can only occur between connected individuals. Each infected individual *i* is assigned *Z*_*i*_ secondary infections drawn from a negative binomial distribution (Fig. 1D), with transmission times sampled from the probability distribution defined by *p*(*t*). These predetermined transmission events are then evaluated against the network topology and intervention status.

Disease progression (Fig. 1C) follows stochastic transitions between compartments, with individuals moving from latently infected (*E*) to either symptomatic (*I*_*S*_) or asymptomatic (*I*_*A*_) infectious states after an incubation period, then to either recovered (*R*) or isolated (*Q*) states depending on testing outcomes. Testing strategies modify transmission by identifying and isolating infectious individuals when *VL*(*t*) exceeds the assay-specific LOD, preventing transmission events scheduled during the isolation period (Fig. 1E).

The simulation advances in discrete time steps, *dt*, updating all individual states, evaluating testing criteria, and executing or preventing transmission events based on current intervention status. This produces epidemic trajectories from which we calculate outcome metrics, including outbreak probability, epidemic size, and testing costs.

### Testing strategies

We investigated three testing strategies used to monitor and control the spread of COVID-19:

1. Daily screening: In this testing scenario, a fixed group of individuals is tested for infection every day. This strategy focuses on regular testing of a predetermined population to detect and isolate cases early, even before symptoms develop. As a result, asymptomatic and pre-symptomatic infectious individuals could be identified a few days after the infection.
2. Symptom-based testing: Symptom-based testing targets individuals who have developed symptoms of the disease, typically around day 5.1 post-infection. Since this strategy relies on the identification of symptoms as a trigger for testing, it cannot detect asymptomatic or pre-symptomatic carriers.
3. Contact-based testing. Contact-based testing involves testing individuals who have been in close contact with a confirmed positive case in the contact network. If any individuals in the contact network get a positive test result, their contacts within the network will be tested for seven consecutive days since the primary case is confirmed. The seven-day testing period covers the pre-transmission of the secondary cases. Note that the contact-based testing strategy alone cannot detect the initially infected individuals; therefore, symptom-based testing with a 75% chance was used in conjunction when the simulations started.

### Testing assays

In our study, we considered the use of different testing assays, including real-time polymerase chain reaction (RT-PCR), reverse transcription-loop-mediated isothermal amplification (RT-LAMP), and antigen test kit (ATK). Each of these assays possesses distinct characteristics, making them well-suited for different applications within the testing strategies. Key differences among these assays are the turnaround time, the time delayed since the testing and the result, the limit of detection (LOD), which is the minimum viral load that the assay can detect, and its accuracy. Our modeling framework focused primarily on the differences in turnaround time and LOD. When the viral load level exceeds the LOD, we assumed a 100% probability of a positive test result. The specific values of turnaround time, limit of detection, and cost per test for each assay are listed in Table 2. Although RT-LAMP and ATK are known to provide results in the order of minutes in practice, we set their turnaround times to zero for modeling simplicity.

**Table 2:** Turnaround time, limit of detection (LOD), and cost per test of each testing assay

	Turnaround time (days)	Limit of detection (copies/mL)	Cost per unit test (USD)	Sources
RT-PCR	1	1 × 10^3^	78.19	[54]
RT-LAMP	0	3 × 10^3^	6.98	[55]
ATK	0	1 × 10^5^	1.19	[26]

### Estimating the probability of a major epidemic

The epidemic from stochastic SIR model can be categorized into a minor outbreak or a major outbreak. The minor epidemic has shorter duration and substantially fewer cases than a major epidemic [56]. The probability of a major epidemic serves as a key metric for evaluating the effectiveness of various testing strategies. Effective testing strategies should minimize the risk of a widespread outbreak. To quantify the risk, we established a threshold value of expected cumulative cases at 1,000 at the end of the 300-day simulation. This threshold value serves as a critical point of distinction between two scenarios. One is the scenario in which the primary infectious individual spreads the disease to only a limited number of cases, and the outbreak eventually goes extinct also called a minor epidemic, and the other is where the disease spreads extensively to a substantial part of the community or a major epidemic. The probability of an major epidemic is then calculated as the ratio of simulation runs that the disease spreads to a substantial part of the community to the total number of runs conducted [57]. The probability of a major epidemic was estimated using 100 batches of simulation, each batch containing 500 model realizations. The duration of both minor and major epidemics was defined as the time from the start of the simulation until the number of infected individuals reached zero.

### Estimating the cost associated with each testing strategy

To estimate the cost of testing, we focused on the number of isolated individuals as a key factor. The cost calculation varies based on the testing strategy employed. For the daily screening strategy, the cost of testing for each simulation was iteratively calculated, starting with an initial cost and increasing based on the number of individuals tested and the number of new cases detected in each simulation. The testing cost is calculated until the end of the outbreak when no infected individuals are left. The cost calculation is more straightforward for the symptom-based testing and contact-based testing strategies. It is determined by multiplying the number of tested individuals by the cost of the assay per test. We then compute the average cost of testing across all simulation runs, focusing on those where at least one infectious individual was tested. This ensures that approximated cost reflects the average testing expenditure per member of the population under each testing strategy, rather than the literal cost incurred by one person taking a test.

## Results

### Impact of daily screening on outbreak dynamics

**Fig. 2:**
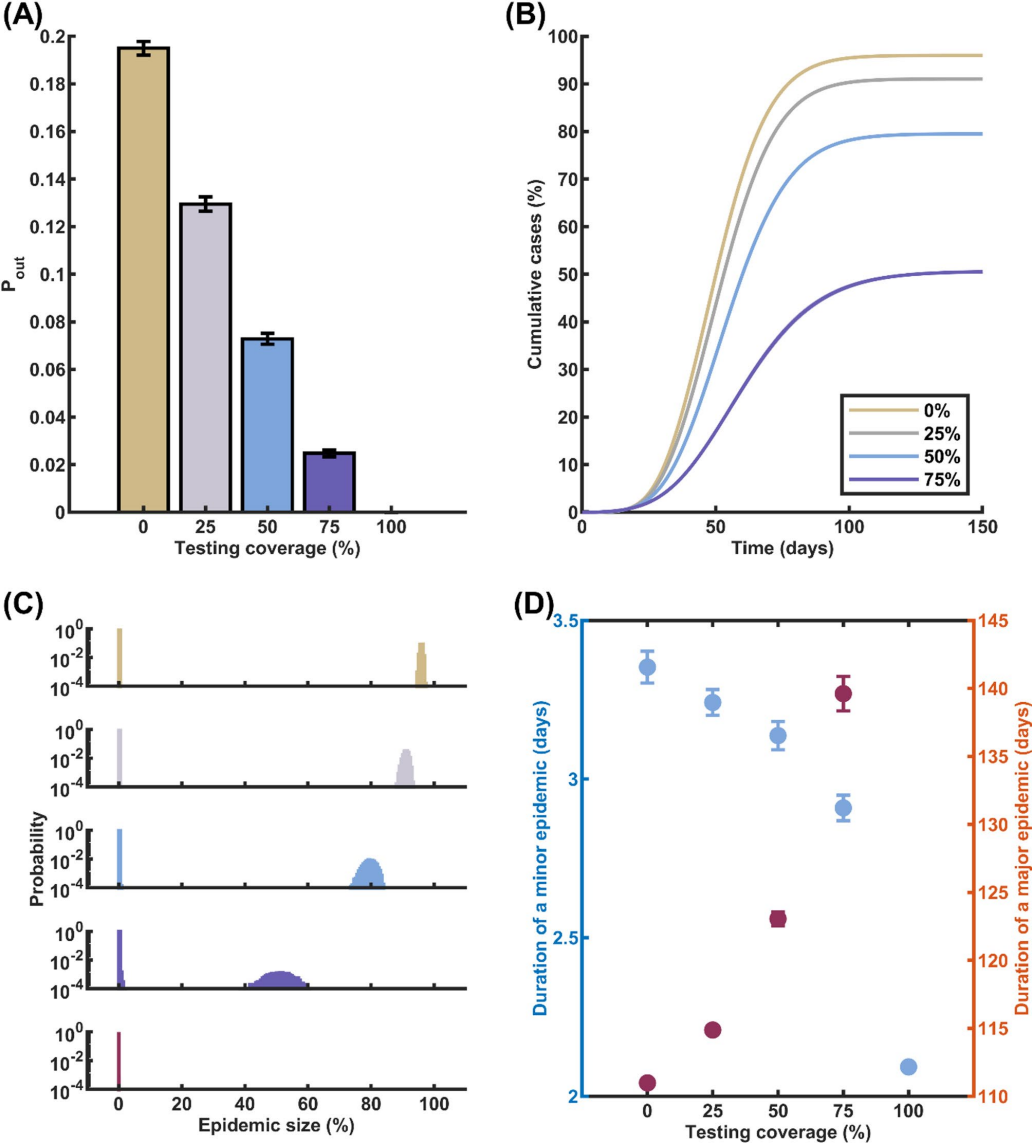
Impact of Daily Screening on Outbreak Dynamics. **A** Probability of a major epidemic (P_out_) as a function of the proportion of the population undergoing daily screening using RT-PCR. As the percentage of individuals screened daily increases, the likelihood of a major epidemic decreases substantially. **B** Expected cumulative COVID-19 cases when a major epidemic occurs, plotted for different proportions of the population (0%, 25%, 50%, and 75%) undergoing daily RT-PCR screening. Higher screening rates lead to a significant reduction in the total number of cases during a major epidemic. **C** Histogram displaying the distribution of epidemic sizes across multiple simulation runs. The histogram shifts towards smaller epidemic sizes as the proportion of the population screened daily increases. **D** Comparison of the average duration of a minor epidemic (blue circles, left axis) and the average duration of a major epidemic(red circles, right axis) for various daily screening rates. Increasing the proportion of the population screened daily results in shorter duration of a minor epidemic, and longer duration in case of a major epidemic

To evaluate the impact of daily screening on COVID-19 transmission, we conducted simulations within a network of 10,000 individuals. Initially, we randomly selected one person to be in the latent state. Our simulations revealed that as the percentage of the tested population increased, there was a decrease in the probability of an outbreak, and the expected cumulative number of cases was smaller in the event of a successful outbreak. Specifically, when 25%, 50%, and 75% of the population underwent daily screening using RT-PCR, the probability of a major epidemic decreased from 0.1912 in the absence of testing to 0.1295, 0.0729, and 0.0248, respectively (Fig. 2A). When all individuals were screened daily, the probability of a major epidemic vanished. In scenarios with the same fraction of individuals undergoing testing, the mean epidemic sizes in the event of a major epidemic were 91.0%, 79.5%, and 50.7%, respectively, compared to 96.0% when there was no testing (Fig. 2B). Moreover, as the percentage of the tested population increased, we observed a wider deviation in epidemic size around its mean (Fig. 2C). The average duration of minor epidemic, when more people participated in the screening, was slightly shortened. Conversely, in case of a major epidemic, the time for the disease to die out was observed to be delayed by approximately a month when 75% of individuals underwent daily screening (Fig. 2D).

We further investigated the impact of the limit of detection and turnaround time associated with the testing assays on the epidemic burden. In cases where an outbreak occurred, daily screening of 25% and 50% of the population using any assay did not result in a significant difference in epidemic size. However, these testing levels did influence the probability of a major epidemic. Notably, daily screening using RT-PCR and RT-LAMP demonstrated comparable performance in terms of the probability of a major epidemic. In contrast, the use of ATK on a daily basis led to a higher probability of a major epidemic compared to the other assays. For instance, when 75% of the population underwent daily screening, the probabilities of an outbreak were 0.0248, 0.0246, and 0.0403 for RT-PCR, RT-LAMP, and ATK, respectively (**Fig. S3** in the Supplementary Information). These results suggest that the choice of testing assay, particularly in terms of LOD and turnaround time, can have a significant impact on the effectiveness of daily screening in mitigating the risk of COVID-19 outbreaks.

### Effectiveness of Symptom-Based testing strategies

While the daily screening strategy has demonstrated remarkable effectiveness in curbing disease transmission by reducing the likelihood of a major epidemic and significantly diminishing the size of epidemics, its implementation requires a substantial volume of testing, leading to high associated costs. This financial burden may pose challenges for widespread adoption and long-term sustainability. In light of these considerations, we sought to investigate an alternative approach: a testing strategy in which tests are conducted exclusively when symptomatic individuals manifest their symptoms.

**Fig. 3:**
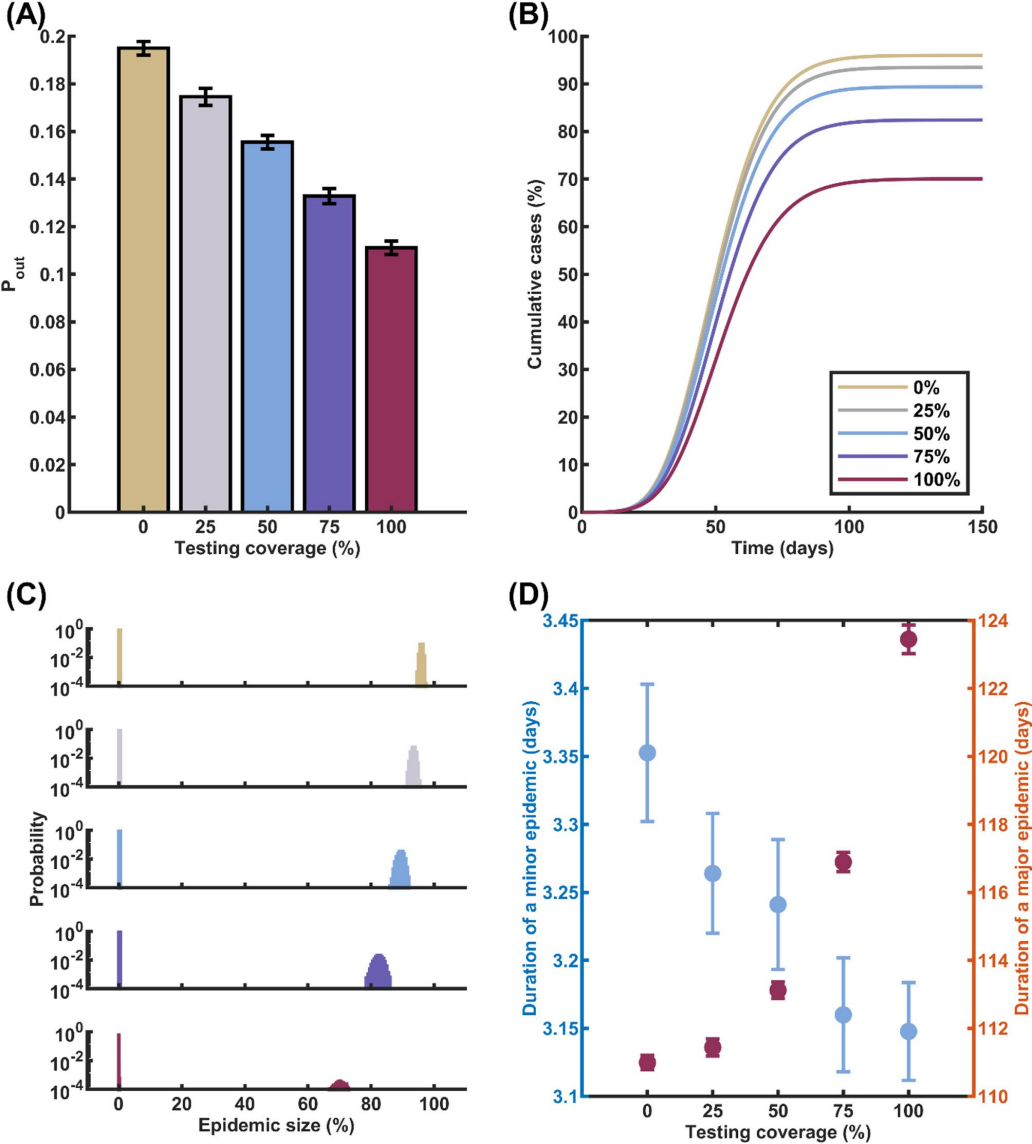
Effects of Symptom-Based Testing on COVID-19 Transmission Dynamics. **A** Probability of a major epidemic as a function of the percentage of symptomatic individuals undergoing RT-PCR testing. **B** Expected cumulative cases in the event of a major epidemic, with varying RT-PCR testing percentages: 0%, 25%, 50%, 75%, and 100%. **C** Histogram illustrating the distribution of epidemic sizes across multiple simulation runs. **D** Comparison of the average duration of a minor epidemic (blue circles, left axis) and the average duration of a major epidemic in the case of successful disease propagation (red circles, right axis) for various symptom-based testing rates

Our findings revealed that conducting tests on a larger portion of symptomatic individuals in the community can effectively decrease the probability of a major outbreak and reduce the epidemic size in the event of a major outbreak (Fig. 3A and B) Interestingly, the outcomes of the symptom-based testing strategy closely mirror those patterns observed in the daily screening strategy. Specifically, when using RT-PCR testing on 25%, 50%, 75%, and 100% of symptomatic individuals, the probabilities of a major epidemic were found to be 0.1846, 0.1555, 0.1328, and 0.1112, respectively (Fig. 3A), compared to the baseline scenario probability of 0.1912 without any testing. Correspondingly, the epidemic sizes, when testing the same proportions of the symptomatic population, were 93.5%, 89.4%, 82.4%, and 70.0%, respectively (Fig. 3B). Furthermore, the results for the duration of both minor and major epidemics exhibited a similar trend to that observed in the daily screening strategy (Fig. 3C and D). These findings suggest that symptom-based testing, when conducted on a sufficient proportion of symptomatic individuals, can be an effective alternative to daily screening in terms of reducing major epidemic probability and mitigating epidemic size.

To further evaluate the impact of different testing assays on epidemic control, we conducted additional simulations with varying LOD and turnaround times associated with RT-PCR, RT-LAMP, and ATK. Our analysis focused on a scenario in which 75% of symptomatic infectious individuals were tested. The results demonstrated that the choice of testing assay had a notable influence on the probability of a major epidemic. When using RT-PCR, RT-LAMP, and ATK assays, the probabilities of a major epidemic were found to be 0.1328, 0.1097, and 0.1161, respectively. This suggests that RT-LAMP provides the lowest probability of a major epidemic among the three assays, followed closely by ATK and then RT-PCR. Similarly, the corresponding epidemic sizes were 82.4%, 79.3%, and 80.0% for RT-PCR, RT-LAMP, and ATK, respectively (Fig. S4 in the Supplementary Information).

### Impacts of integrating Contact-Based testing with Symptom-Based strategies

Contact-based testing has emerged as another widely adopted approach during the COVID-19 epidemic. This strategy involves identifying and testing individuals who have been in close contact with confirmed infected cases. In our research, we investigated the impact of incorporating contact-based testing as a supplementary measure alongside the primary symptom-based testing approach. Specifically, we examined a hybrid strategy where the main testing method was symptom-based, with 75% of symptomatic infectious individuals being tested using the ATK assay. Upon obtaining a positive test result from a symptomatic individual, the strategy triggered a contact tracing process within the contact network. All close contacts of the confirmed case were then subjected to daily testing for seven consecutive days, commencing as soon as the primary case’s positive test result was available. By testing the contacts promptly and repeatedly over a week, this approach aimed to quickly identify and isolate any secondary infections resulting from exposure to the primary case. The seven-day testing period was chosen to cover the potential incubation period and pre-symptomatic transmission window of COVID-19.

**Fig. 4:**
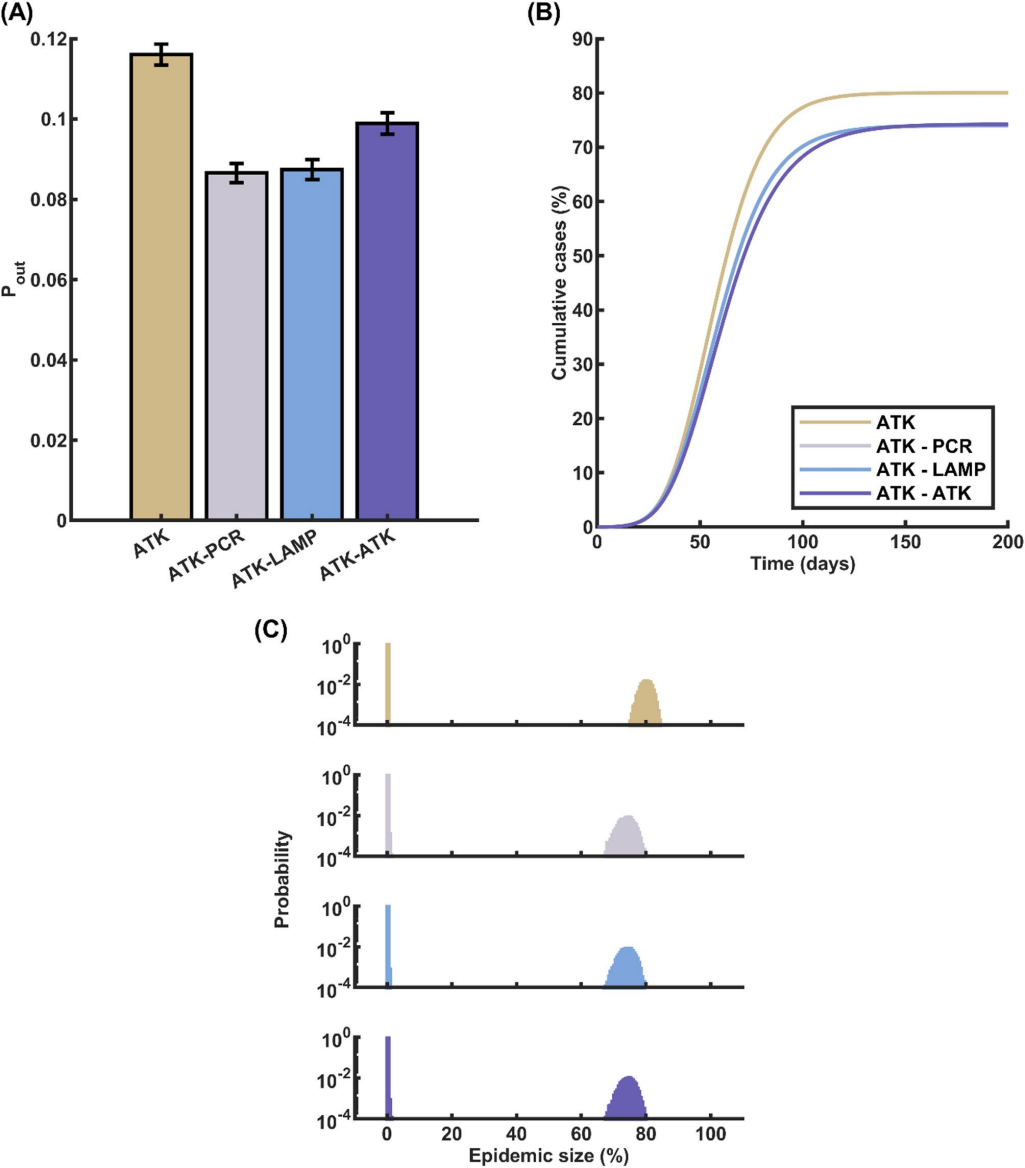
Effects of Combining Contact-Based Testing with Symptom-Based Testing on COVID-19 Outbreak Dynamics. **A** Probability of a major epidemic when incorporating contact-based testing alongside symptom-based testing. The baseline scenario represents symptom-based testing with 75% of symptomatic individuals tested using ATK. The addition of contact-based testing using RT-PCR, RT-LAMP, or ATK for all contacts of confirmed cases results in a notable decrease in the likelihood of an outbreak compared to the baseline. **B** Expected cumulative COVID-19 case count in the event of a major epidemic for the baseline scenario and the three contact-based testing strategies. The incorporation of contact-based testing leads to a consistent reduction in the total number of cases during an outbreak, regardless of the testing method used for contacts. **C** Histogram of epidemic sizes for different contact-based testing approaches. The four panels, from top to bottom, represent contact-based testing using ATK alone, ATK for symptom-based testing and RT-PCR for contact-based testing, ATK for symptom-based testing and RT-LAMP for contact-based testing, and ATK for both symptom-based and contact-based testing. The histograms demonstrate that the inclusion of contact-based testing shifts the distribution of epidemic sizes towards lower values, indicating better containment of outbreaks

The results of our simulations demonstrated that the incorporation of contact-based testing alongside symptom-based testing led to a substantial reduction in both the probability of an outbreak and the expected cumulative number of cases. In the baseline scenario, where only symptom-based testing with ATK was employed, the probability of a major epidemic was 0.1161. However, when additional testing was conducted on all close contacts of confirmed cases, the major epidemic probabilities decreased slightly. Specifically, the probabilities dropped to 0.0865, 0.0874, and 0.0989 when the contacts were tested using RT-PCR, RT-LAMP, and ATK assays, respectively (Fig. 4A). This finding highlights the effectiveness of contact tracing and testing in identifying and isolating potential secondary infections, thereby reducing the overall transmission risk. Interestingly, despite the notable reduction in major epidemic probability, the expected cumulative number of cases in the event of a major epidemic remained relatively consistent at around 74%, irrespective of the testing method used for close contacts (Fig. 4B and C). This suggests that while contact-based testing is effective in preventing outbreaks altogether, it may have a limited impact on the final size of an epidemic once it takes hold. Nevertheless, the decreasing in major epidemic probability underscores the value of integrating contact-based testing into the overall testing strategy to enhance COVID-19 containment efforts.

### Cost-Effectiveness analysis of testing strategies for outbreak control

To evaluate the cost-effectiveness of each testing strategy, we conducted a comprehensive analysis of the associated expenses. Our findings revealed that, among all the strategies considered, daily screening incurred the most significant costs. This can be attributed to the repetitive nature of daily testing and the potential inefficiencies arising from its frequent implementation. However, it is crucial to acknowledge that daily screening demonstrated remarkable effectiveness in identifying infectious individuals before they exhibit symptoms or remain asymptomatic, which is a key advantage in controlling the spread of the virus.

**Fig. 5:**
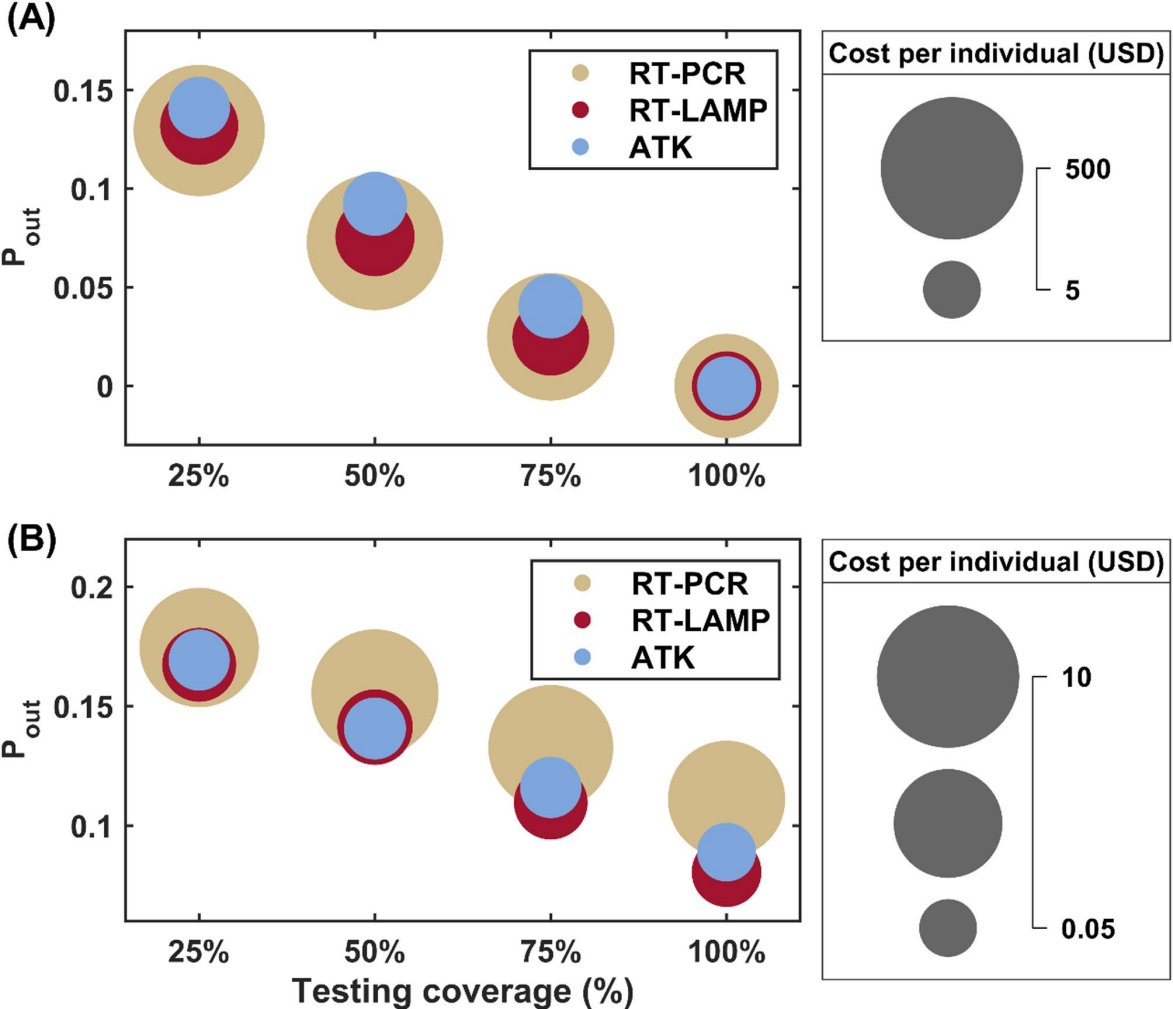
Comparison of Major Epidemic Probabilities and Testing Costs for Different Strategies and Assays. **A** Probability of a major epidemic under daily screening strategies with varying percentages of the population tested (25%, 50%, 75%, and 100%) using RT-PCR, RT-LAMP, and ATK assays. The circle sizes represent the logarithmic scale of the associated testing costs per individual in USD. **B** Probability of a major epidemic under symptom-based testing strategies with different proportions of symptomatic individuals tested (25%, 50%, 75%, and 100%) using the three assays. The marker colors indicate the type of assay used: brown for RT-PCR, red for RT-LAMP, and purple for ATK. The figure illustrates the trade-off between reducing a major epidemic probability and testing costs for each strategy and assay combination

The cost of controlling an outbreak through daily screening varied depending on the percentage of the population tested and the testing assay employed. When using RT-PCR for daily screening, the costs per individual were found to be 392, 448, 355, and 164 USD for testing 25%, 50%, 75%, and 100% of the population, respectively (Fig. 5A). Notably, the costs were comparatively lower when utilizing RT-LAMP and ATK testing methods for daily screening. However, it is important to consider that ATK testing carried a slightly higher risk of outbreaks compared to RT-PCR and RT-LAMP, highlighting the trade-off between cost and effectiveness.

In contrast, symptom-based testing emerged as a more cost-effective approach. When employing RT-PCR for symptom-based testing, the costs were approximately 5.6, 7.0, 6.6, and 5.2 USD per individual (Fig. 5B) for testing 25%, 50%, 75%, and 100% symptomatic cases, while ATK testing costs were even lower, in the order of 0.08, 0.10, 0.09, and 0.06 USD per individual (Fig. S5) with the same percentage as mentioned earlier. These findings suggest that symptom-based testing offers a more economically viable option for outbreak control, particularly when resources are limited.

### Impact of population immunity on outbreak risk and testing strategies

Epidemic transmission dynamics vary significantly across different phases of a pandemic and are strongly influenced by population immunity levels. In the early stages, when the entire population is predominantly susceptible, the introduction of a single infected individual can trigger a widespread outbreak. As the pandemic progresses, population immunity accumulates through natural infection and vaccination, reducing overall transmission potential.

Despite increasing immunity, localized outbreaks can still occur when community immunity levels remain below the herd immunity threshold, or new cases are continuously introduced from external sources. To simulate these later-phase scenarios, we implemented a model that accounts for pre-existing population immunity. We initialized our simulations with various proportions of the population (ranging from 30% to 80%) already in the recovered state, representing individuals with acquired immunity through previous infection or vaccination. This approach allowed us to model the epidemic dynamics typical of later pandemic phases.

Unlike our earlier simulations that began with a single index case, we incorporated a continuous importation mechanism in these scenarios, introducing one new infectious individual per day throughout the 300-day simulation period. This methodological approach simulates the condition of ongoing case importation that communities face during established pandemics, particularly in areas with high mobility or travel connections.

By combining pre-existing immunity with continuous case importation, we created a modeling framework to evaluate how different testing strategies perform when implemented in communities with partial protection but ongoing exposure risk. This approach allowed us to assess which testing strategies remain effective and cost-efficient during later epidemic phases, providing guidance for long-term public health planning beyond initial outbreak control.

**Fig. 6:**
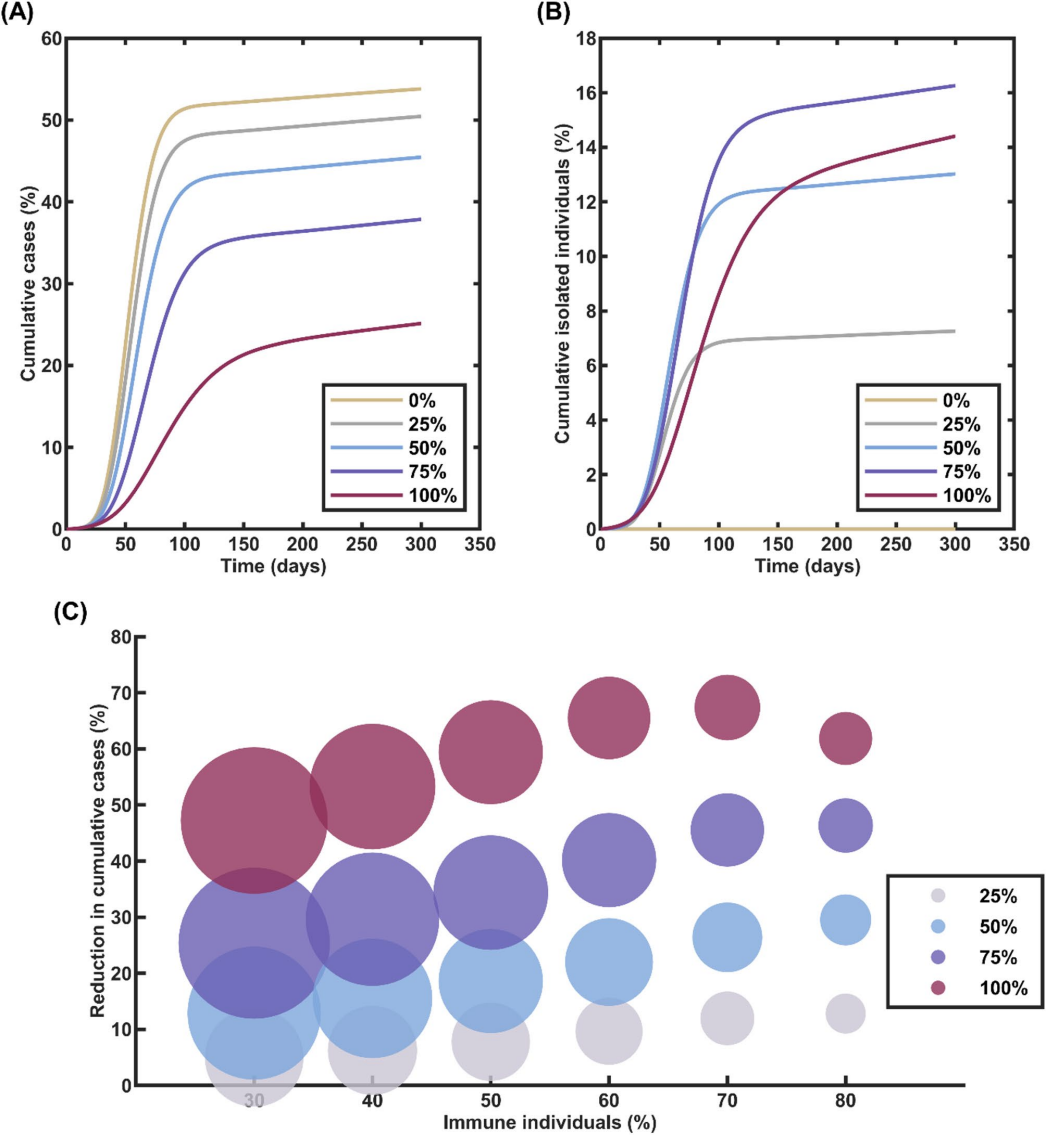
Effectiveness of Symptom-Based Testing in Scenarios with Continuous Importation of Infectious Individuals and Varying Levels of Population Immunity. **A** Proportion of expected cumulative COVID-19 cases when different percentages of symptomatic individuals (25%, 50%, 75%, and 100%) undergo testing, and 40% of the community has already acquired immunity. Increasing the percentage of symptomatic individuals tested leads to a substantial reduction in the proportion of expected cumulative cases, even in the presence of continuous importation of infections. **B** Proportion of individuals isolated under different symptom-based testing percentages when 40% of the community is immune. Higher testing rates result in a larger proportion of individuals being isolated, contributing to the containment of the outbreak. **C** Percentage reduction in expected cumulative COVID-19 cases for various proportions of symptomatic individuals undergoing testing, plotted against the proportion of the population already immune. The size of the circles represents the extent of testing utilized, with larger circles indicating higher numbers of tests employed

In our analysis, we implemented a symptom-based testing strategy and varied the testing percentage from 25% to 100% to assess its impact on outbreak dynamics in the presence of population immunity. Our results were analyzed using the results from 500 simulation runs at the final date of our simulation, which is day 300. Our findings revealed that symptom-based testing, even with varying levels of prior population immunity, can significantly reduce the proportion of expected cumulative cases. In the absence of testing, the proportion of expected cumulative cases in the scenario with 40% initial immunity was 53.79%. However, as the percentage of symptomatic infectious individuals being tested increased, the proportion of expected cumulative cases decreased accordingly. Specifically, when 25%, 50%, 75%, and 100% of symptomatic infectious individuals were tested, the proportion of expected cumulative cases reduced to 50.43%, 45.44%, 37.85%, and 25.12%, respectively (Fig. 6A). In parallel, the percentage of isolated individuals increased to 7.259%, 13.02%, 16.26%, and 14.41% when 25%, 50%, 75%, and 100% of symptomatic infectious individuals were tested, respectively (Fig. 6B).

Finally, our analysis revealed a relationship between the percentage of symptomatic individuals tested and the overall testing counts. Surprisingly, we found that testing 100% of symptomatic individuals required a similar amount of testing resources as testing only 50% of them. In fact, we found that when the initial immune percentage was 50%, 60%, or 70%, testing 100% of symptomatic infectious individuals required slightly lower testing quantities compared to testing 50% of them while simultaneously achieving a greater reduction in expected cumulative cases (Fig. 6C).

## Discussion

In this study, we conducted a comprehensive evaluation of various testing strategies for managing COVID-19 outbreaks, focusing on daily screening, symptom-based testing, and contact-based testing. Each of these strategies was assessed using different testing assays, including RT-PCR, RT-LAMP, and ATK, either alone or in combination. Our simulations demonstrated that daily screening was the most effective approach in mitigating the risk of a COVID-19 outbreak and reducing its overall impact once it occurred. By increasing the proportion of individuals undergoing regular testing, the likelihood of community transmission significantly decreased. However, daily screening also emerged as the most costly option among the strategies evaluated due to the high frequency of testing required.

Our findings offer valuable insights for different public health decision-making contexts. When the primary objective is to prevent an outbreak following case introduction, daily screening of at least 75% of the population using RT-PCR or RT-LAMP provides the most effective approach, reducing the probability of a major epidemic from 0.1912 to approximately 0.025. However, this approach incurs substantial costs (164–392 USD per individual) and may be most appropriate when resources are abundant or when delaying transmission is critical, such as during vaccine development. For decision-makers prioritizing cost-effective disease burden reduction, symptom-based testing of all symptomatic individuals combined with contact tracing offers the best balance. This approach reduces outbreak probability to approximately 0.09–0.10 while keeping costs below 0.10 USD per individual when using ATK. This strategy is particularly valuable in resource-limited settings or during the later stages of a pandemic. When population immunity is already present and the goal is to minimize further disease burden during continuing case importation, our results indicate that testing all symptomatic individuals is both the most effective and surprisingly cost-efficient approach, challenging the intuitive assumption that higher testing coverage necessarily requires proportionally higher investment.

To mitigate expenses, the use of the ATK assay, which is the least expensive option, could be considered. It is important to note, however, that the ATK assay has a higher limit of detection (LOD) compared to RT-PCR, which may lead to delayed virus detection and potentially missed cases. One way to balance the cost and effectiveness of daily screening could be to reduce the testing frequency to intervals such as every 3, 5, or 7 days as suggested in Larremore et al. [35]. This approach could help manage costs while still maintaining a reasonable level of outbreak control. Furthermore, our analysis suggests that RT-PCR and RT-LAMP assays outperform ATK in terms of reducing outbreak risk and infection rates. This advantage is likely due to their lower LOD and faster virus detection capabilities, enabling earlier identification and isolation of infected individuals.

Symptom-based testing is a commonly adopted strategy, owing to its lower cost compared to daily screening, despite its reduced effectiveness in mitigating outbreak risks and limiting epidemic size. Our analysis reveals that even when 100% of symptomatic infectious individuals are tested, the lowest achievable probability of a major epidemic saturates at approximately 0.13. This limitation highlights the inherent challenges of relying solely on symptom-based testing, as it fails to identify asymptomatic and pre-symptomatic cases, which can contribute significantly to disease transmission [8, 58, 59]. However, the cost-effectiveness of symptom-based testing makes it an attractive option, particularly in resource-constrained settings. Interestingly, our study suggests that the LOD of the testing assay is not the primary determinant of its efficacy in mitigating the disease burden. Instead, the turnaround time of symptom-based testing emerges as a crucial factor in reducing outbreak risk and infection rate. This finding emphasizes the importance of rapid testing and timely isolation of infected individuals, as delays in obtaining test results can lead to further transmission events.

Our modeling results also demonstrated that implementing additional testing on the contacts of primary cases can help reduce the risk of a major epidemic and the overall epidemic size, though these reductions were modest. By identifying and isolating infected individuals within the contact network, the chain of transmission can be interrupted, contributing to limiting the spread of the virus. It’s worth noting that these modest benefits were observed even under our model’s optimistic assumptions of perfect contact identification and immediate testing, suggesting that real-world contact tracing effectiveness may face additional challenges. Interestingly, while the epidemic size remained similar across different assays when a successful outbreak occurred, both RT-PCR and RT-LAMP assays outperformed ATK in terms of reducing the overall risk of an outbreak. This superior performance can be attributed to the lower LOD of RT-PCR and RT-LAMP assays, which allows for earlier detection of the virus in infected individuals. The faster detection enables prompt isolation measures to be implemented, thereby minimizing the opportunity for further transmission. In contrast, the higher LOD of ATK may lead to delayed detection and, consequently, a higher chance of an infected individual spreading the virus before being identified and isolated.

The duration of both minor and major epidemics when daily screening and symptom-based testing share the same trends. The longer duration of a major epidemic with higher testing coverage suggests that increased testing slows disease transmission, effectively flattening the curve but also prolonging the outbreak. However, this should be considered alongside the reduced outbreak risk and smaller epidemic size. In contrast, for a minor epidemic, higher testing coverage helps cut transmission chains more quickly, leading to faster containment for both daily screening and symptom-based testing.

In addition to assessing testing strategies in the early stages of an outbreak, we also investigated scenarios where a portion of the population has already acquired immunity to the disease. This situation is particularly relevant in the later stages of an epidemic when a significant number of individuals may have been infected and recovered or have received vaccinations. In these scenarios, we focused on the symptom-based testing strategy, which is commonly employed during the later phases of an epidemic [60]. Our analysis revealed that the most effective and cost-efficient approach is to test all symptomatic infectious individuals, as this allows for the identification and isolation of active cases, thereby limiting further disease transmission.

Additionally, our analysis reveals an interesting observation: when focusing on population-level epidemic control, testing all symptomatic infectious individuals required fewer total tests than testing 75% of symptomatic cases in scenarios with existing population immunity. This counterintuitive finding suggests that the relationship between testing coverage and resource utilization is not always proportional, particularly when considering total disease burden reduction across an entire population. However, it’s important to recognize that this cost-effectiveness assessment depends significantly on the decision-maker’s perspective and priorities. For public health authorities focused on minimizing overall epidemic size and transmission, this finding supports testing all symptomatic individuals. In contrast, different stakeholders—such as hospital administrators—might prioritize other outcomes like reducing absolute case numbers in the short term or minimizing healthcare system burden, which could lead to different optimal testing strategies.

However, relying solely on symptom-based testing may not be sufficient to break the transmission chain completely, as it does not account for asymptomatic or pre-symptomatic individuals who can still spread the virus [49, 58]. To address this limitation, we could imply from our first study scenario that implementing contact-based testing as a complementary measure can further reduce the transmission chain. By identifying and testing the contacts of confirmed cases, regardless of their symptomatic status, we can detect and isolate infected individuals who may have been missed by symptom-based testing alone. This combined approach of symptom-based and contact-based testing proves to be a potent strategy for controlling the spread of the disease, even in situations where a significant proportion of the population has already acquired immunity.

However, our study has several important limitations that should be acknowledged when interpreting the results. Firstly, we did not account for the possibility of reinfection, which may occur several months after the initial infection [61]. Reinfection could potentially alter the dynamics of disease transmission and the effectiveness of testing strategies, particularly in the later stages of an epidemic when immunity begins to wane. Secondly, our testing approach assumes a deterministic outcome based on viral load and the assay’s limit of detection, without considering the inherent variability in assay sensitivity and specificity observed in real-world settings [25]. This simplification may overestimate the effectiveness of testing strategies, as false-negative results could allow infectious individuals to remain in the community, and false-positive results could unnecessarily isolate non-infectious individuals. Thirdly, we did not incorporate the heterogeneity of viral load at the individual level, which may influence the detectability of infections and the effectiveness of testing approaches [62]. Individual variation in viral shedding, including potential superspreading events, could significantly impact transmission dynamics in ways not captured by our model. Fourthly, our model relies on several strong assumptions that may lead to overestimation of testing effectiveness. For example, we assume symptomatic individuals are not isolated in the absence of testing, while in reality, many symptomatic individuals might self-isolate regardless of testing. We also assume isolation is perfectly effective, meaning isolated individuals cannot transmit the disease, which is unlikely in household or healthcare settings. Fifth, we assume perfect compliance with both testing and isolation. Real-world compliance varies with factors including testing frequency (daily screening may induce fatigue), symptom severity (symptomatic individuals more likely to comply), and socioeconomic constraints (ability to afford isolation). Reduced compliance would decrease effectiveness while potentially altering cost-effectiveness rankings between strategies. Additionally, we model no delay between symptom onset and testing—once tested, individuals are immediately isolated—which neglects real-world logistical constraints.

Furthermore, initiating testing at the start of the simulation when only one person is exposed may be an unrealistic assumption. Our sensitivity analysis where testing is triggered when 1% of the population is exposed shows that when testing coverage is insufficient, the probability of a major epidemic approaches 1 (Fig. S6), highlighting how the timing of testing implementation is critical for outbreak control. Our study assumes a specific contact network structure based on a power-law distribution, which may not fully capture the complexity and diversity of real-world social interactions, including clustering effects, temporal variations, and context-specific contact patterns [63]. While our sensitivity analysis on networks with larger mean degree shows similar trends (Fig. S7 and Fig. S8), other network properties might influence disease transmission dynamics and the effectiveness of testing strategies.

Our study utilized a population size of 10,000 individuals, which is smaller than many real-world communities facing epidemic control decisions. While our additional simulations suggest the general trends remain consistent across different population sizes (Fig. S9), several scale-dependent factors should be considered when extrapolating our findings. Implementing testing strategies in larger populations would likely face additional logistical challenges and resource constraints that scale non-linearly. The absolute number of cases in larger outbreaks could exceed healthcare capacity thresholds, creating additional consequences beyond what our proportional metrics indicate. Larger populations also typically exhibit more complex spatial structuring and mobility patterns that might influence transmission dynamics and testing effectiveness.

Finally, our study does not consider the impact of viral variants with different transmission characteristics, incubation periods, or clinical presentations, which could affect the relative effectiveness of the testing strategies evaluated. The emergence of variants with higher transmissibility or immune escape properties might necessitate adjustments to testing approaches beyond what our model predicts.

These limitations highlight the need for cautious interpretation of our results when applying them to real-world epidemic control decisions. Our modeling provides valuable insights into the relative effectiveness and cost-efficiency of different testing strategies, but implementation should be tailored to specific contexts and adapted as new evidence emerges.

## Conclusions

In conclusion, our study provides a comprehensive assessment of various testing strategies for managing COVID-19 outbreaks, considering both their effectiveness and cost-efficiency. Our model suggests daily screening may be the most robust approach to minimizing the likelihood and impact of outbreaks, but it comes with the highest financial burden, necessitating strategic and targeted implementation. Our evaluation of RT-PCR, RT-LAMP, and ATK assays highlights that tests with lower limits of detection and also those with faster turnaround times could improve outbreak control. However, this improved performance often comes at an increased expense. ATK and RT-LAMP present more cost-effective alternatives, albeit with a slightly reduced efficacy compared to RT-PCR. Symptom-based testing and contact-based testing emerge as economically viable options for disease management, particularly when used in combination. These strategies prove especially effective in curtailing transmission during the later stages of an epidemic when a significant portion of the population has acquired immunity.

## Supplementary Information

Supplementary Material 1

## Data Availability

All key data generated or analyzed during this study are included in this published article and its supplementary information file. The complete simulation code used to generate these results is available on GitHub at [https://github.com/hmpbtrev/Testing\_code](https:/github.com/hmpbtrev/Testing_code).
