# Supplementary material for: Modeling the effectiveness of RT-PCR, RT-LAMP, and antigen testing strategies for COVID-19 control
Source: BMC Infect Dis. 2025 Oct 17;25:1351. doi: 10.1186/s12879-025-11793-7 (PMC12535133; doi:10.1186/s12879-025-11793-7)
Supplement: Supplementary file 1 — Supplementary Material 1 [file 12879_2025_11793_MOESM1_ESM.pdf]

Supplemental Information:
Modeling the effectiveness of RT-PCR, RT-LAMP, and antigen testing strategies for COVID-19 control

Tanakorn Chantanasaro[1,2], Chayanin Sararat[1,2], Noppamas Yolai[1,2], Pikkanet Suttirat[1,2], Kawin Nawattanapaiboon[3,4], Somchai Chauvatcharin[5], Sudarat Chadsuthi[6], and Charin Modchang[1,2,7,8*]

[1]Biophysics Group, Department of Physics, Faculty of Science, Mahidol University, Bangkok 10400, Thailand

[2]Center for Disease Modeling, Faculty of Science, Mahidol University, Bangkok, 10400, Thailand

[3]Zenostic Co., Ltd, Bangkok 10400, Thailand

[4]School of Materials Science and Innovation, Faculty of Science, Mahidol University, Bangkok 10400, Thailand

[5]Department of Biotechnology, Faculty of Science, Mahidol University, Bangkok 10400, Thailand

[6]Department of Physics, Faculty of Science, Naresuan University, Phitsanulok 65000, Thailand.

[7]Centre of Excellence in Mathematics, MHESI, Bangkok 10400, Thailand

[8]Thailand Center of Excellence in Physics, Ministry of Higher Education, Science, Research and Innovation, 328 Si Ayutthaya Road, Bangkok 10400, Thailand

* To whom correspondence should be addressed.

E-mail: charin.mod@mahidol.edu

Innate immune response model

The within-host viral dynamics model based on a target cell limited model tracks the total number of target cells (T), cells refractory to infection (R), cells in the eclipse phase of infection (E), i.e., infected cells not yet reproducing virus, productively infected cells (I), and total viruses (VL), assumed to be proportional to the viruses in nasal swaps.

The differential equations for the innate immune response model are [1]

$$\frac{dT}{dt} = -\beta(VL)T - \emptyset IT + \rho R$$

$$\frac{dR}{dt} = \emptyset IT - \rho R$$

$$\frac{dE}{dt} = \beta(VL)T - kE$$

$$\frac{dI}{dt} = kE - \delta I$$

$$\frac{dVL}{dt} = \pi I - c(VL)$$

where $\emptyset$ is a constant representing the rate that innate signaling makes target cells refractory, and ρ is the rate that refractory cells transition back into target cells. β is a infected rate constant, $1/k$ is eclipse period, δ is death rate of infected cells, π is a composite parameter for virus production and sampling, and c is the virus clearance rate.

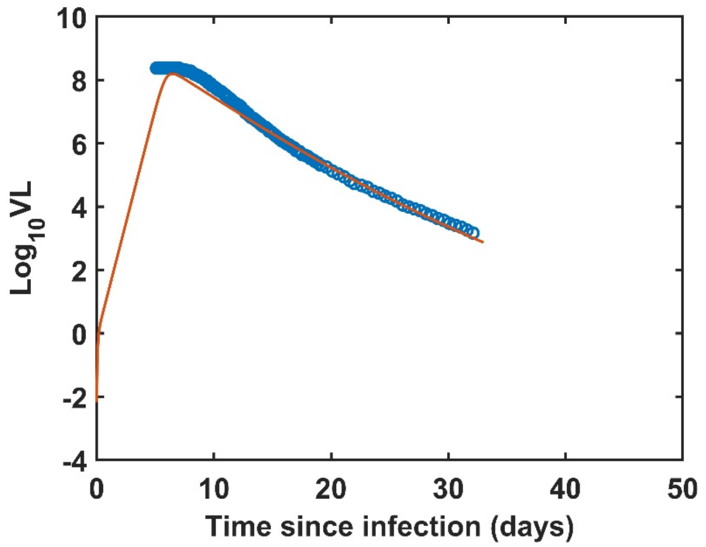

Figure S1. Plot of within-host viral dynamics model fit to the data. The fitted parameters for the innate immune response model were obtained with a NMSE of 0.029, where $\beta = 3.3710 \times 10^{-8}$, $\delta = 0.5432$, $\pi = 34.8702$, $\emptyset = 10.1086 \times 10^{-8}$, and $\rho = 0.9999$.

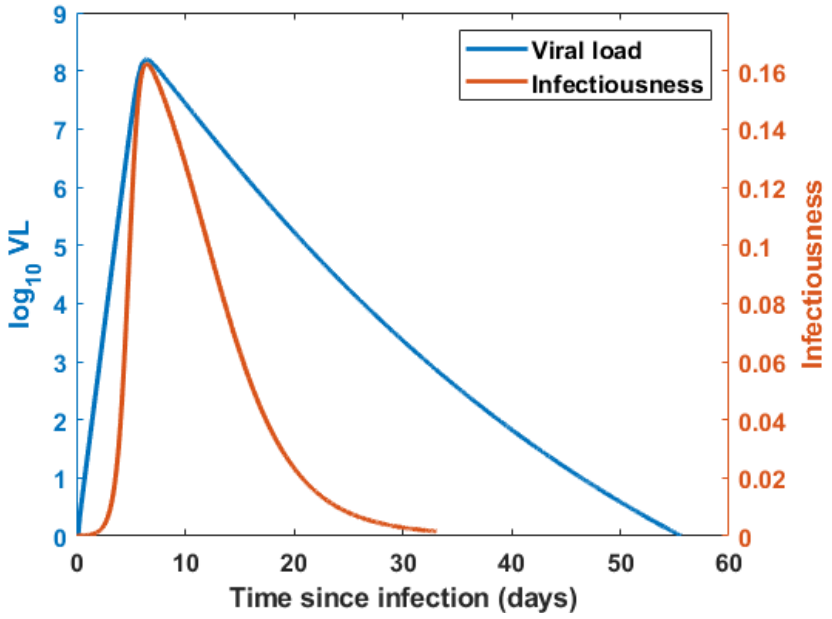

Figure S2. **Plot of estimated viral load and infectiousness profile**. (Blue line) The estimated viral load from fitting is assumed to be the same for all infected individuals and

is used in testing whether the tested result from individual is positive or not. (Red line) The infectiousness profile derived from the viral load indicates the transmissibility of infected individual during their course of infection.

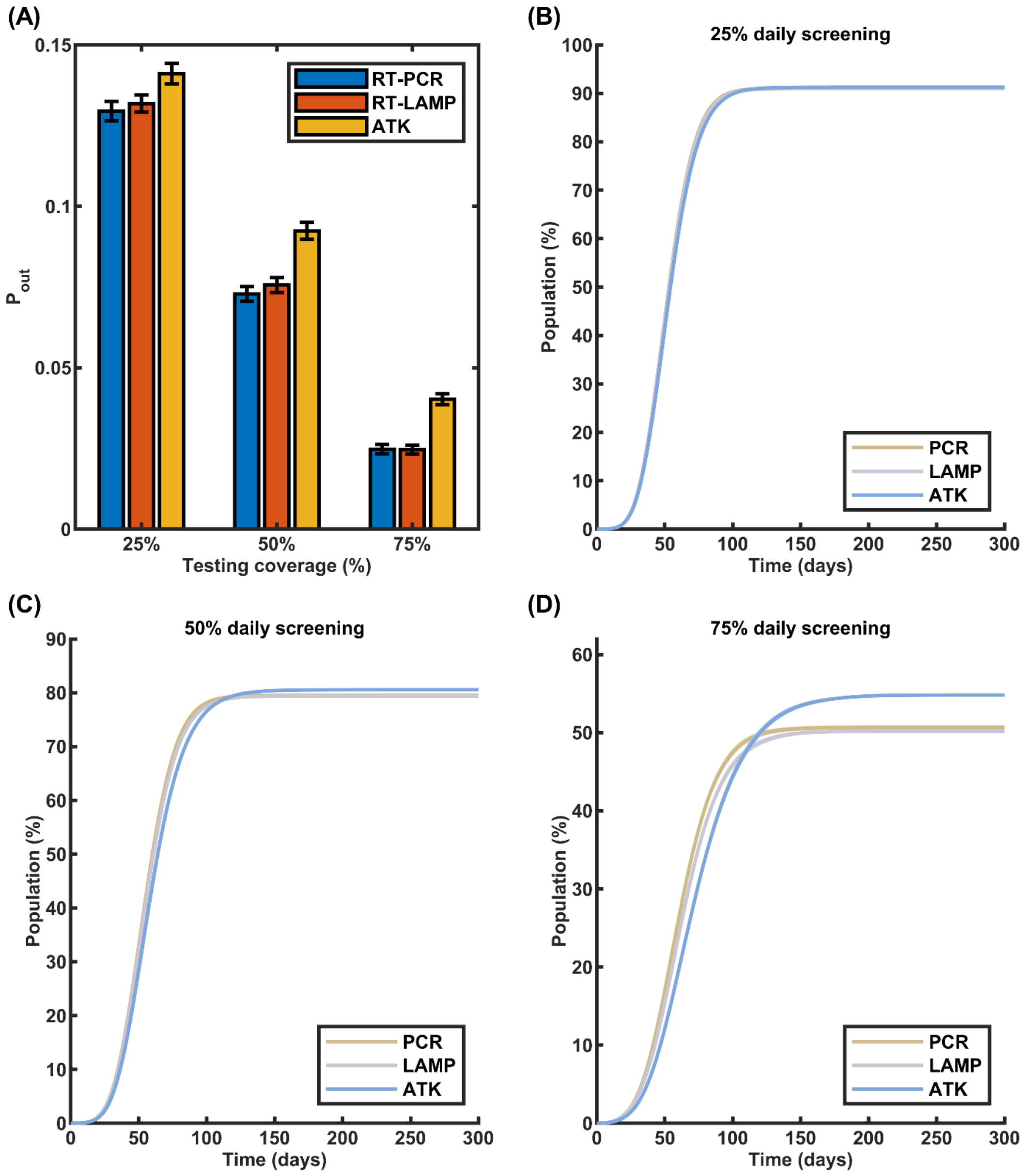

Figure S3. Impact of limit of detection (LOD) and turnaround time on the effectiveness of daily screening strategies. (A) Probability of an outbreak occurring under different daily screening scenarios using RT-PCR, RT-LAMP, and ATK assays. **(B)**

– **(D)** Cumulative COVID-19 cases when an outbreak occurs, with 25%, 50%, and 75% of the population undergoing daily screening, respectively. The choice of testing assay, characterized by its LOD and turnaround time, influences the effectiveness of daily screening in reducing outbreak risk and controlling epidemic size.

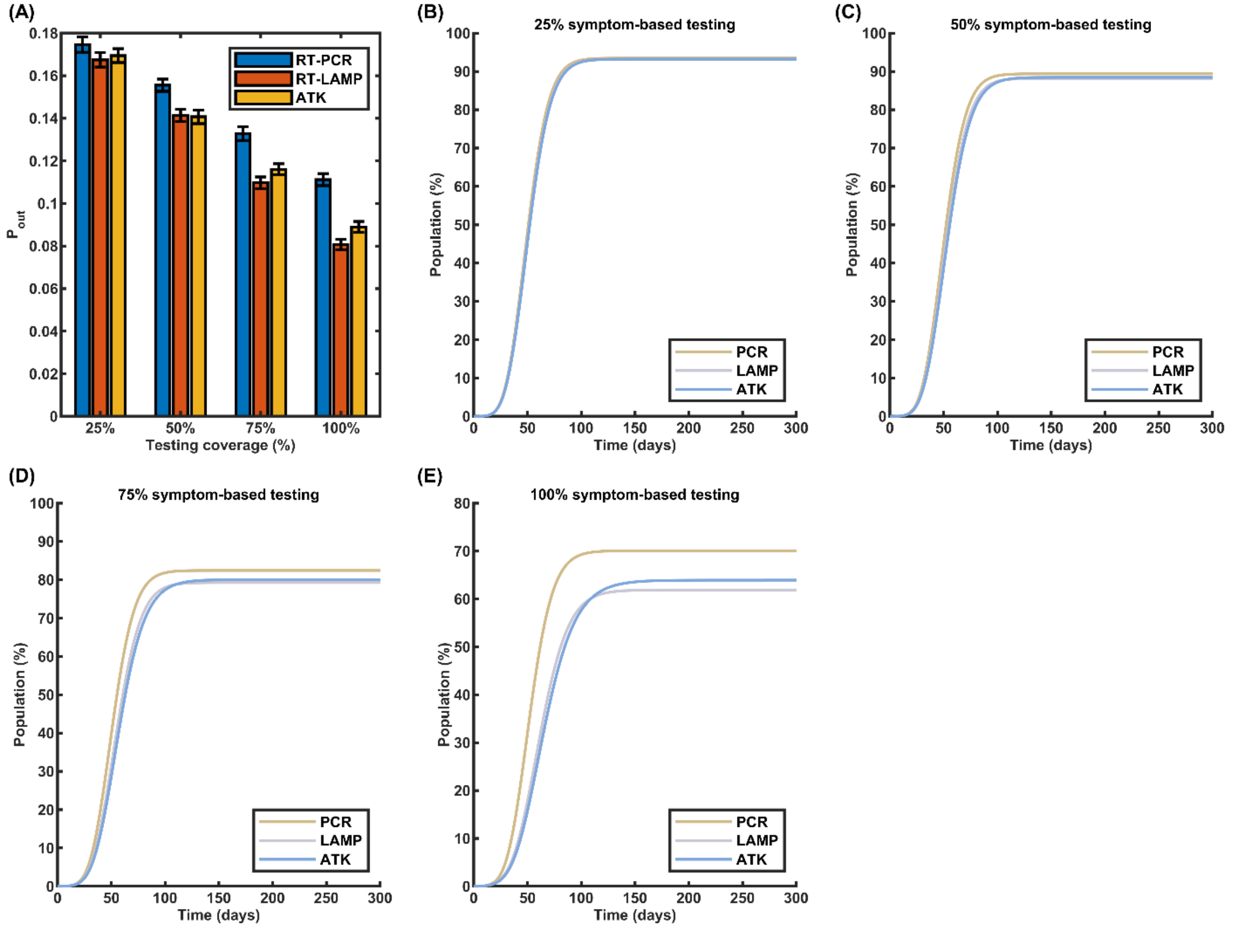

Figure S4. Impact of limit of detection (LOD) and turnaround time on the effectiveness of symptom-based testing strategies. (A) Probability of an outbreak occurring when different proportions of symptomatic individuals (25%, 50%, 75%, and 100%) are tested using RT-PCR, RT-LAMP, and ATK assays. **(B) – (E)** Cumulative COVID-19 cases in the event of a successful outbreak, with varying percentages of the symptomatic population undergoing testing: 25%, 50%, 75%, and 100%, respectively. The results demonstrate how the choice of testing assay, based on its LOD and

turnaround time, affects the efficacy of symptom-based testing in mitigating outbreak risk and reducing the overall disease burden.

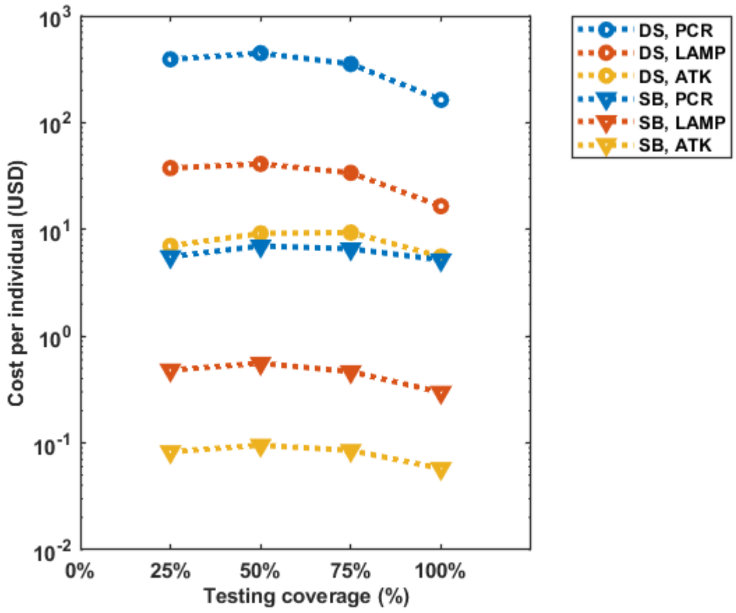

Figure S5. **Plot of cost per individual used under different testing strategies and testing assays**. The estimated cost per individual used for different testing strategies. The circle marker indicates the daily screening, and the triangle indicates the symptom-based testing. The colors represent the testing assay. Blue, red, and yellow refer to testing by RT-PCR, RT-LAMP, and ATK respectively.

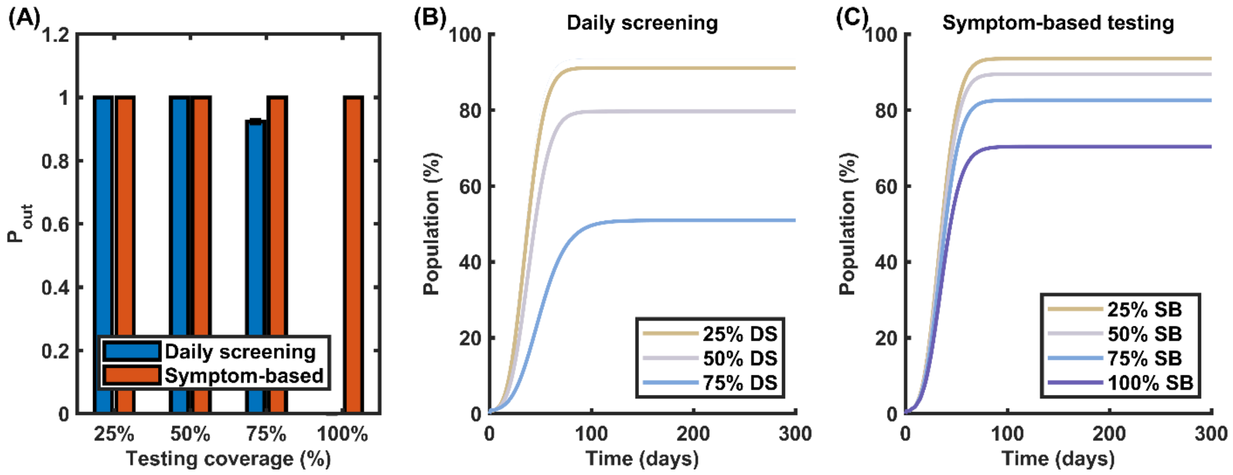

Figure S6. The probability of a major epidemic and the expected cumulative cases in the event of a major epidemic under RT-PCR testing, when testing is initiated after 1% of the population has been exposed. (**A**) Probability of a major epidemic as a function of the percentage of testing coverage by daily screening and symptom-based testing. (**B**) Expected cumulative cases in the event of a major epidemic, with varying RT-PCR daily screening percentages: 0%, 25%, 50%, and 75%. (**C**) Expected cumulative cases in the event of a major epidemic, with varying RT-PCR symptom-based testing percentages: 0%, 25%, 50%, 75%, and 100%.

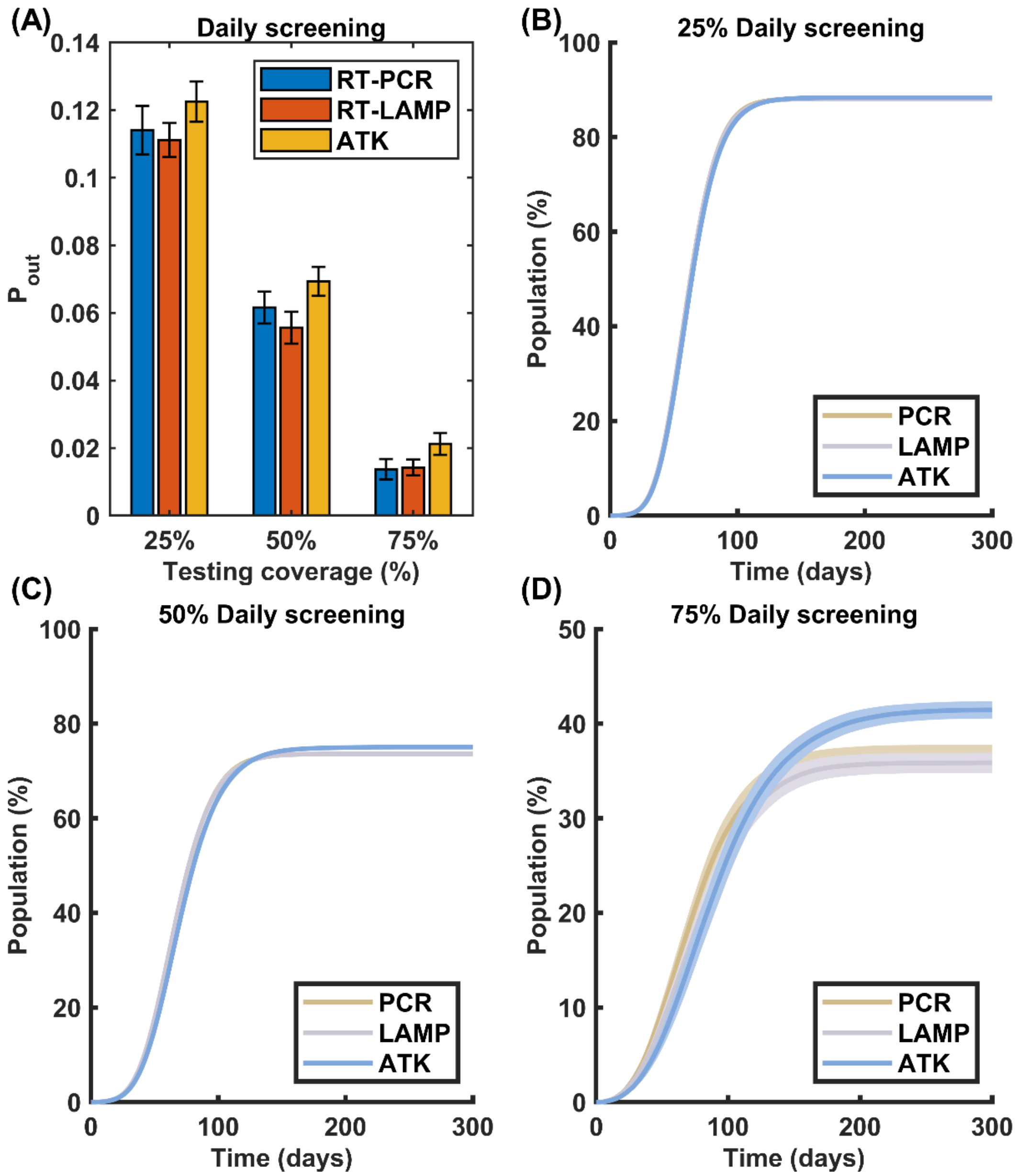

Figure S7. The probability of a major epidemic and the expected cumulative cases in the event of a major epidemic under daily screening in contact network with mean degree 68. (A) The probability of a major outbreak shares the same trend with the result from contact network with mean degree 34. **(B) – (D)** Cumulative COVID-19 cases in the event of a successful outbreak, with varying percentages of population undergoing daily screeing: 25%, 50%, and 75%, respectively. The results show the same trend with the result from the contact network with mean degree 34.

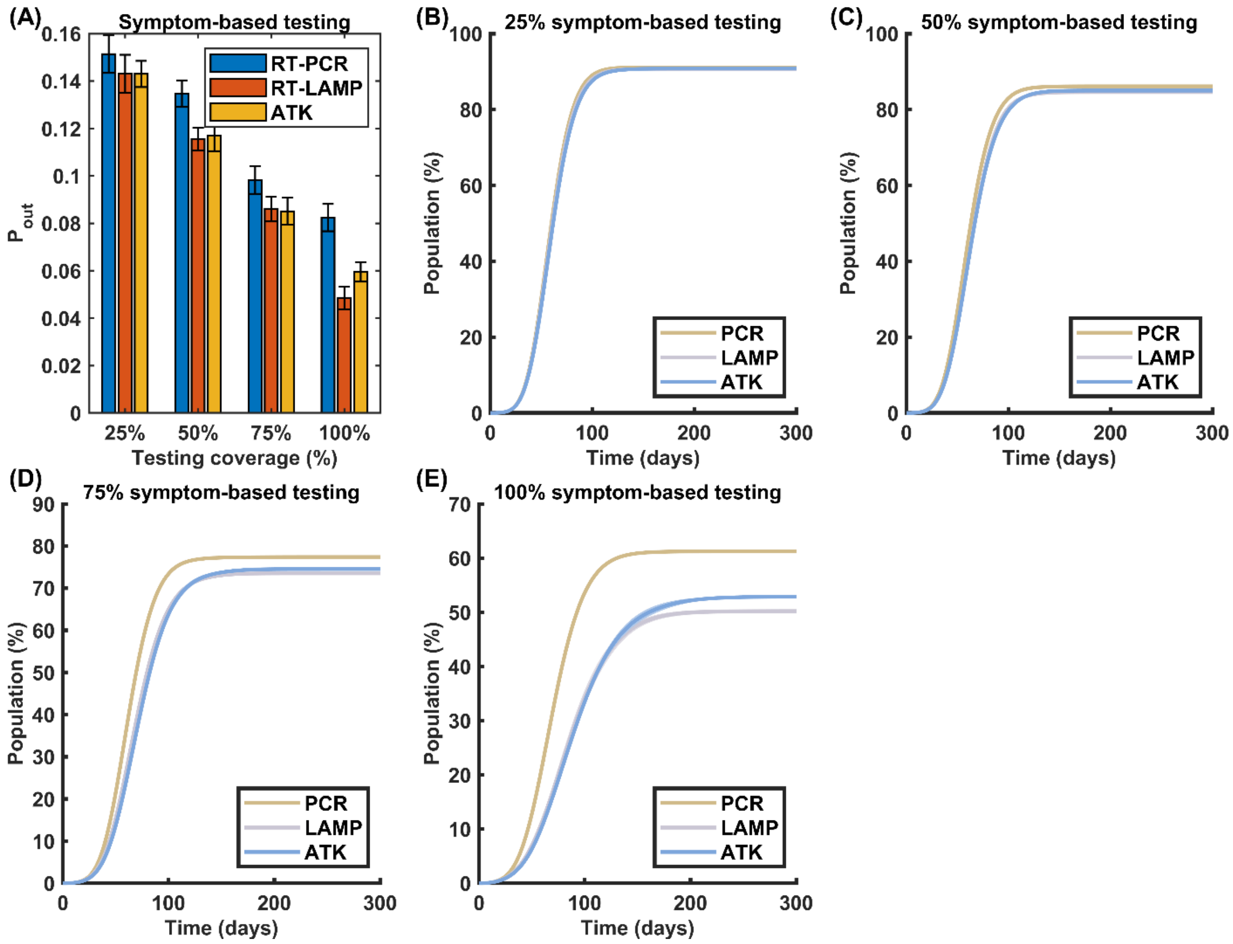

Figure S8. The probability of a major epidemic and the expected cumulative cases in the event of a major epidemic under symptom-based testing in contact network with mean degree 68. (A) The probability of a major outbreak shares the same trend with the result from contact network with mean degree 34. **(B) – (E)** Cumulative COVID-19 cases in the event of a successful outbreak, with varying percentages of the symptomatic population undergoing testing: 25%, 50%, 75%, and 100%, respectively. The results demonstrate a similar trend with the result from the contact network with mean degree 34.

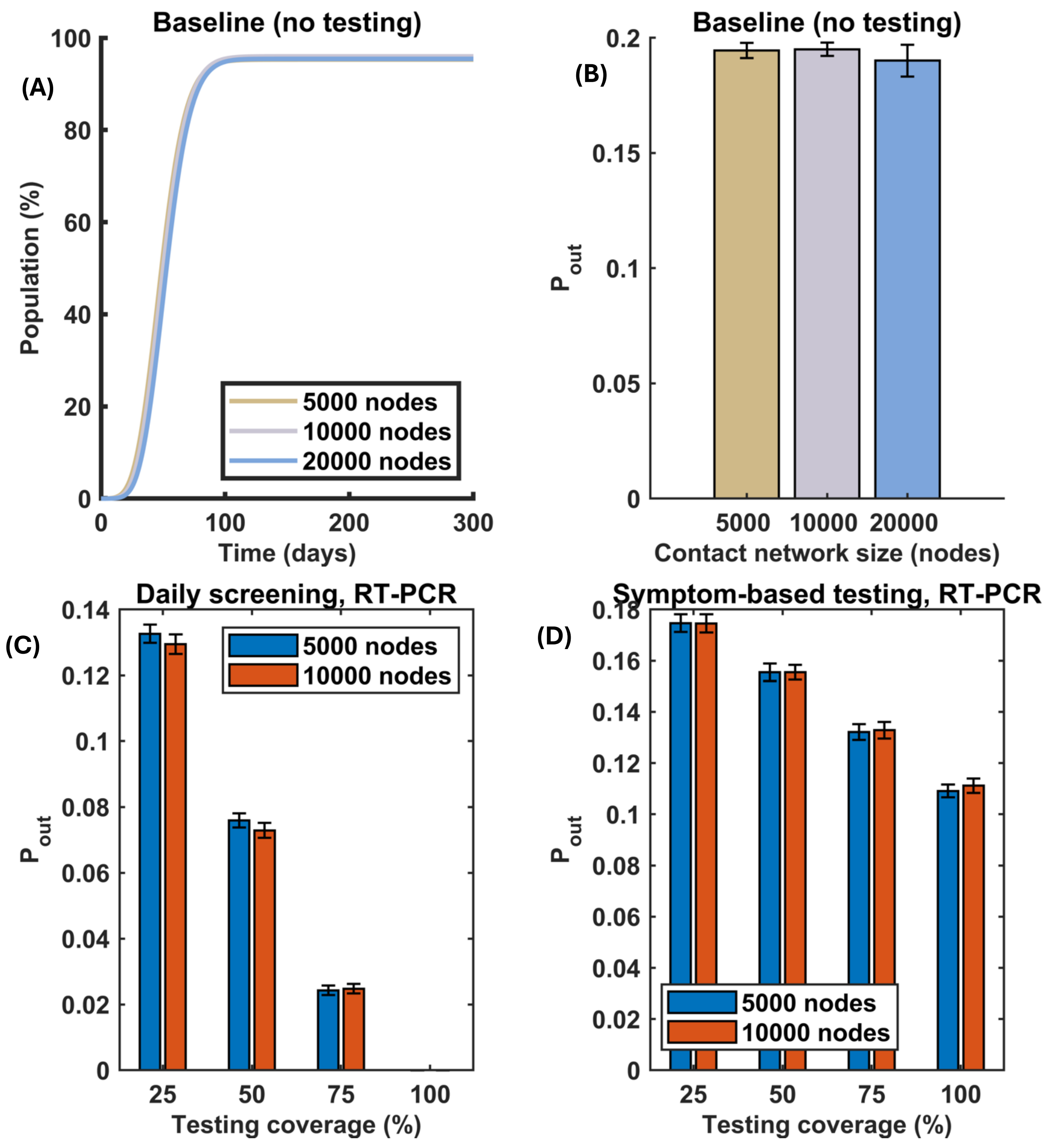

Figure S9. Comparison of epidemic dynamics and testing effectiveness across different population sizes. (A) Epidemic curves showing the percentage of infected population over time for networks with 5,000, 10,000, and 20,000 nodes under baseline conditions (no testing). **(B)** Probability of a major epidemic (P_{out}) in baseline scenarios (no testing) for different population sizes, showing consistent values across network sizes. **(C)** Probability of a major epidemic for different testing coverages (25%, 50%, 75%,

100%) under daily screening with RT-PCR, comparing networks with 5,000 and 10,000 nodes. **(D)** Probability of a major epidemic for different testing coverages (25%, 50%, 75%, 100%) under symptom-based testing with RT-PCR, comparing networks with 5,000 and 10,000 nodes. Error bars represent standard error. Results demonstrate that epidemic dynamics and the relative effectiveness of testing strategies remain consistent across different population sizes, supporting the generalizability of our findings.

Reference:

1. Ke R, Zitzmann C, Ho DD, Ribeiro RM, Perelson AS: **In vivo kinetics of SARS-CoV-2 infection and its relationship with a person's infectiousness**. *Proc Natl Acad Sci U S A* 2021, **118**(49).
